# Recent Perspectives on APP, Secretases, Endosomal Pathways and How they Influence Alzheimer’s Related Pathological Changes in Down Syndrome

**DOI:** 10.4172/2161-0460.S7-002

**Published:** 2013-03-20

**Authors:** Boris DeCourt, William Mobley, Eric Reiman, Raj Jatin Shah, Marwan N Sabbagh

**Affiliations:** 1 Banner Sun Health Research Institute, Sun City AZ, USA; 2 University of California-San Diego, San Diego CA, USA; 3 Banner Alzheimer’s Institute, Phoenix AZ, USA

**Keywords:** Down syndrome, Trisomy, Over-expression

## Abstract

Down syndrome is one of the most common genetic conditions occurring in one in 700 live births. The trisomy of chromosome 21 causes over-expression of APP which in turn is indicated in the increased production of Aβ associated with AD. This makes DS the most common presenile form of AD exceeding PS1 and PS2 FAD. Since a majority of DS individuals develop dementia, it is important to examine whether DS and sporadic AD share common features, for example, to anticipate shared treatments in the future. Here we explore commonalities and differences for secretases and endosomal pathways in DS and AD.

## Introduction

Down syndrome (DS) is a disorder caused by partial to complete trisomy of chromosome 21. It is characterized by several developmental abnormalities, including craniofacial abnormalities, microcephaly, developmental delays, cardiac defects (Atrial Septal Defects and Ventricular Septal Defects), hypothyroidism, and cataracts among others, and appears to be a risk factor for the subsequent development of Alzheimer’s disease (AD) [1]. Patients with Down Syndrome (DS) have an extremely high incidence of early onset AD [2,3]. Virtually all DS patients develop the characteristic plaques and tangles by the age of 35, with a gradual increase in the rate of AD between ages 40 and 60 years, to 50% prevalence [4]. These grim statistics mark DS as the richest genetic source of AD and explain why AD is one of the most feared outcomes for people with DS and their families. In spite of these concerns, very little basic research and translational activities have been directed at DS.

The mechanism by which DS leads to fibrillar Aβ deposition is related to the extra copy of the amyloid precursor protein (APP) gene located on the distal arm of chromosome 21, which is present in three copies in DS. In DS, triplication of chromosome 21 invariably includes the *APP* gene (21q21) encoding the amyloid precursor protein (APP), which is suggested to increase APP expression and lead to cerebral accumulation of APP-derived amyloid-beta peptides (Aβ), early-onset AD neuropathology, and age-dependent cognitive sequelae [5]. What is mysterious is why with such heavy Aβ burdens by age 40 it often takes another 15 to 20 years or more years for individuals with DS to begin showing symptoms of dementia, and why others are able to escape the clinical manifestation of dementia. Some argue that age of onset and reduced longevity may underlie the lack of complete penetrance of the dementia phenotype. Others speculate that aneuploidy might contribute to lack of complete penetrance.

Although the exact molecular mechanisms leading to AD in individuals with DS (referred to as DSAD) are poorly understood, parallels have been drawn between DS and AD brain pathology. In this review, we consider a few recent discoveries suggesting that DSAD have several neuropathological characteristics of sporadic AD.

## Amyloid Pathology

### APP and Aβ plaques

Virtually all individuals with DS have the senile plaques and neurofibrillary tangles characteristic of AD by the age of 40 [6]. One of the neuropathological hallmarks of AD is the abundance of dense senile plaques in the brain composed of aggregated Aβ peptides, principally 40 and 42 amino acid in length (Aβ40 and Aβ42). In the past two decades, it has been shown that Aβ is produced via sequential cleavage of APP by β- and γ-secretases. Historically, the mapping of *APP* gene to chromosome 21 was made using DS samples [7]. The *APP* gene is found in the DS obligate region, and the protein is frequently overexpressed in the adult DS brain [8]. It is commonly believed that APP gene dosage results in increased amounts of Aβ and extracellular plaque formation in the DS brain, and that this process begins early in life [9] and further, soluble Aβ peptide build up precedes plaque formation in DS [10]. Interestingly, several familial forms of AD have been linked to mutations in the APP gene [11] - most of which surrounding the β-secretase cleaving site - that increase the production of Aβ and results in early manifestation of the dementia. These findings support the idea that the *APP* gene found on the third 21 chromosome in DS likely plays a crucial role in the development of amyloid pathology in individuals with DS [12]. For example, in 1996 Lemere et al. found that brain Aβ deposition starts as early as age 12 but dense Aβ40 deposits were not detected until age 30, i.e. when degenerating neurites around plaques are first observed [9]. They also observed that Aβ42 immunoreactivity was always higher than Aβ40 at any given age. Furthermore, a study of DS brains from subjects ranging 3–73 years of age showed that several anti-Aβ42 antibodies induced strong intraneuronal signals in very young DS patients (i.e. age 3–4), but this signal intensity declined as extracellular Aβ plaques gradually accumulated and matured [13]. Combined with other findings, this underscores that amyloid deposition is an early and possibly seminal event in the pathogenesis of the dementia in the setting of DS. However, much is still to discover about the cerebral changes occurring during the conversion from DS to DSAD.

Recently, new brain imaging techniques have made possible the visualization of Aβ deposits in the living brain. In a study of 9 subjects with DS and 14 controls, all of whom underwent Pittsburgh Compound B (PiB) PET imaging, it was observed that all DS cases over age 45 had increased PiB activity, suggestive of higher amyloid loads [14]. Interestingly, in a case report study, Sabbagh et al. successfully used Florbetapir F18 to visualize Aβ deposits in a single DS patient with AD, then correlated imaging findings to autopsy findings [15]. Thus, while it is possible that fibrillar Aβ PET ligands underestimate plaque (particularly diffuse plaque) deposition, these results indicate that most individuals with DSAD display a buildup of dense senile plaques, which is very similar to sporadic AD. Moreover, these data suggest that minimally invasive *in vivo* imaging techniques could be used to detect and track aggregated Aβ both in individuals with DS and subjects at risk to develop AD, which could help evaluate the effect of Aβ-modifying treatments when such therapeutics becomes available.

### Secretases

As explained above, amyloidogenesis results from the consecutive proteolytic processing of APP by β- and γ-secretases [16]. Briefly, β-secretase cleaves the luminal domain of APP, close to the plasma membrane, releasing a large extracellular fragment referred to as soluble APPβ (sAPPβ) into the milieu. The remaining APP fragment, consisting of both a transmembrane and intracellular domains (called C99 fragment), is then processed by γ-secretase that cuts C99 in the middle of the transmembrane domain to release Aβ into the extracellular milieu and the APP intracellular domain (AICD) into the cytoplasm. In an alternative, non-amyloidogenic pathway α-secretase cleaves APP in the middle of the Aβ region (between Lys-16 and Leu-17 of Aβ), releasing a large sAPPα domain into the milieu. The remaining APP transmembrane and intracellular domains (named C83) are further processed by γ-secretase to release AICD and a non-amyloid peptide called p3.

Strong genetic and pharmacological evidence revealed that the main β-secretase in the brain is β amyloid cleaving enzyme 1 (BACE1) [17–21]. It was also proposed that BACE1 is the rate-limiting enzyme in amyloidogenesis [22]. Furthermore, compelling data indicate that BACE1 levels are increased at early stages of AD [23] making this enzyme a potential target for anti-amyloid therapies [24]. However, whether BACE1 levels and activity are increased in individuals with DS is still controversial. On the one hand, it was reported that mature BACE1 isoforms and activity were enriched in DS fibroblasts [25]. On the other hand, BACE1 protein levels and activity were not significantly different in DS versus normal control fetal and adult brain samples [8,26]. Similarly, controversial data have been reported regarding the amyloidogenic potential of BACE2 [25,27], a BACE1 homolog protein this is located on chromosome 21 and which was initially thought to act as a β-secretase but is now believed to work as an α-secretase [28]. Therefore, it would be interesting to investigate further the role of BACE1 and BACE2, for example by measuring their levels and activities, in both DS and DSAD post-mortem brain samples.

Gamma-secretase is as a complex comprising four transmembrane proteins, i.e. presenilin 1/2 (PS1/2), nicastrin (NCT), presenilin enhancer 2 (PEN2), and anterior pharynx-defective 1 (APH1), that assemble into a heteromultimer unit generating and regulating the γ-secretase activity [29–31]. Very little is actually known about γ-secretase in DS, while ample literature exists for AD. However, the acute lowering of Aβ synthesis by pharmacological inhibition of γ-secretase improved learning and memory performance in a mouse model of DS [32]. These data suggest that Aβ might have deleterious effects on cognitive performance in both DS and AD, thus anti-amyloid therapies, including γ-secretase inhibition, would likely benefit both populations. Importantly, however, this treatment would also markedly increased C99 and C83 whose potentially deleterious impact on synaptic structure are now being explored.

### Endosomal Pathology

Early endosomes support the homeostasis, growth, and functions of cells by transporting membrane-associated and extracellular molecules in a pathway that includes a number of endosome types. In neurons, endocytosis plays a crucial role in the physiological ability of neurons to sense and control their surrounding milieu. The system of organelles in the the endocytic pathway (EP) consists of early endosomes, recycling endosomes, late endosomes, and lysosomes. Early endosomes are the first sorting site in the endocytic pathway, in which BACE1 interacts directly with APP [33]. Early endosomes produce C99 and C83 as well as Aβ; they also mediate the Aβ uptake by neurons. Endosomes are transported in neurons to carry out several functions: neurotrophic signaling from synapses to the cell body, delivery of various cargoes to the lysosome for degradation, cargo transport to the golgi apparatus for utilization, and recycling components back to the plasma membrane for further use [34].

In early AD and DS, endosomes are enlarged [35]. A number of consequences can be envisioned as a result of failure to normally traffic enlarged endosomes, or changes in the cargoes or signals carried by enlarged endosomes: disrupted neurotrophic signaling, abnormal distribution of cargoes targeted for degradation, and changes in the normal trafficking of receptors at synapses. APP is also present in endosomes [36]. Interrupting APP function or processing within endosomes may underlie endosome dysfunction and contribute to the pathogenesis of AD and DSAD. Indeed, because endosomal pathology develops very early both in DS and AD the argument has been made that it plays a role in neurodegenerative processes [37]. It is likely that changes in early endosomes are linked to changes in downstream component of the endocytic pathway, including late endosomes and lysosomes, pointing to alterations in many portions of the endocytic pathway. A recent report attempted to define what it is about *APP* gene dose that is responsible for endocytic dysfunction by examining DS fibroblasts. The data supported the view that C99 and not its Aβ byproducts were key [35].

In sporadic AD, it has been found that the brain contain three times the normal amount of neuronal early endosomes. This is attributed to the increase in activity of the endocytic pathway, causing an increase in early endosomes. Also, previous studies have concluded that a buildup of lysosomes and an increase in gene expression (e.g. Rabs genes) leading to the activation of the lysosome system, are typical neuronal responses to sporadic AD and DS. For instance, Cataldo et al. measured markers of EP activity in DS brains and found that early endosomes were significantly enlarged in pyramidal neurons as early as 28 weeks of gestation- i.e. decades before classical AD neuropathology develops [38]. This response cannot be solely attributed to Aβ overproduction or deposition because this response starts in DS before Aβ is detected, and fails to develop in familial AD even though Aβ is overproduced and deposited in large amounts [39].

Early involvement of the endocytic pathway is of great interest due to the fact that the early endosome provides a point of convergence within the cell for many key etiological factors in AD, including the Aβ peptide. Despite the difficulty in defining post-mortem the status of the endocytic pathway, a number of changes indicate that the EP activity is increased. Firstly, both Rab5 mRNA and protein are upregulated, and experimentally increasing Rab5 levels results in increased size of early endosomes and increased endocytosis [40]. Secondly, the over expression of Rab4 is present suggesting increased activity in recycling of endosomes [41]. Thirdly, effector proteins that regulate the activity of Rab5 impact vesicle membrane fusion and docking thus play a critical role in endocytosis [38]. For example, enlargement of Rab5-positive early endosomes in the AD brain was associated with elevated levels of Rab4 immunoreactivity and translocation of rabaptin 5 to endosomes, implying that both endocytic uptake and recycling are activated. Furthermore, it was reported that endocytic abnormalities in fibroblasts from individuals with DS are reversed by lowering the expression of either APP or BACE1, and that increased APP expression alone in normal disomic cells is sufficient to induce endosomal pathology [35]. These data suggest that regulation of gene expression and epigenetic regulations may occur in the context of increased APP gene dose and expression, though this possibility has as yet received little attention.

## Conclusion

In conclusion, many recent molecular and imaging studies of DS have implicated molecular mechanism that are involved in the development of AD, such as Aβ production, endosomal pathology, and BACE1 activity, suggesting neuropathological parallels between the two conditions. It is commonly accepted that *APP* gene dosage in the DS brain results in increased amounts of Aβ and extracellular plaque formation begins early in life. BACE1 dysregulation potentially represents an overlapping biological mechanism with sporadic AD, as well as common therapeutic targets [28], though no one has explored this possibility in clinical trials yet.

However, the link between the molecular mechanisms summarized here and the development of dementia needs further evaluation. Meantime, advances in the development of investigational anti-amyloid treatments, and the use of fibrillar Aβ PET and other imaging methods in the preclinical detection and tracking of AD are likely going to facilitate studies on DS neuropathology [42,43]. Finally, we strongly believe that DS represents a very good model of FAD-like amyloid pathology and individuals with DS would greatly benefit from anti-amyloid therapies.
